# Epigenetic immunomodulatory effect of eugenol and astaxanthin on doxorubicin cytotoxicity in hormonal positive breast Cancer cells

**DOI:** 10.1186/s40360-021-00473-2

**Published:** 2021-01-28

**Authors:** Mariam A. Fouad, Mohamed M. Sayed-Ahmed, Etimad A. Huwait, Hafez F. Hafez, Abdel-Moneim M. Osman

**Affiliations:** 1 Pharmacology and Experimental Oncology Unit, National Cancer Institute, Cairo University, Cairo, 11796 Egypt; 2 Department of Biochemistry, Faculty of Sciences, King Abdulaziz University, Experimental Biochemistry Unit, King Fahad Medical Research Centre, Jeddah, Saudi Arabia

**Keywords:** Eugenol, Astaxanthin, Doxorubicin, Breast Cancer cells

## Abstract

**Background:**

Hormonal receptor positive (HR+) breast cancer is the most commonly diagnosed molecular subtype of breast cancer; which showed good response to doxorubicin (DOX)-based chemotherapy. Eugenol (EUG) and astaxanthin (AST) are natural compounds with proved epigenetic and immunomodulatory effects in several cancer cell lines. This study has been initiated to investigate the molecular mechanism (s) whereby EUG and AST could enhance DOX cytotoxicity in MCF7 cells.

**Methods:**

Cytotoxic activity of DOX alone and combined with either 1 mM EUG or 40 μM AST was performed using sulphorhodamine-B assay in MCF7 cells. Global histones acetylation and some immunological markers were investigated using ELISA, western blotting and quantitative RT-PCR techniques. Functional assay of multidrug resistance was performed using rhodamine 123 and Hoechst 3342 dyes. Flow cytometry with annexin V and propidium iodide were used to assess the change in cell cycle and apoptosis along with the expression of some differentiation, apoptosis and autophagy proteins.

**Results:**

DOX alone resulted in concentration-dependent cytotoxicity with IC_50_ of 0.5 μM. Both EUG and AST significantly increased DOX cytotoxicity which is manifested as a significant decrease in DOX IC_50_ from 0.5 μM to 0.088 μM with EUG and to 0.06 μM with AST. Combinations of DOX with 1 mM EUG or 40 μM AST significantly increased the level of histones acetylation and histone acetyl transferase expression, while reduced the expression of aromatase and epidermal growth factor receptor (EGFR) when compared with 0.25 μM DOX alone. Also both combinations showed higher uptake of rhodamine but lower of Hoechst stains, along with increased the percentage of caspase 3, and decreased the expression of CK7 and LC3BI/II ratio. EUG combination induced IFγ but reduced TNFα causing shifting of cells from G2/M to S and G0/ G1 phases. Combination of DOX with EUG induced apoptosis through the higher BAX/ BCl2 ratio, while with AST was through the increase in caspase 8 expressions.

**Conclusion:**

EUG and AST potentiated the anticancer activity of DOX through epigenetic histones acetylation along with the immunonomodulation of different apoptotic approaches in MCF7 cells.

## Background

According to the most recent Global Cancer Statistics issued in 2018, breast cancer is the most commonly diagnosed cancer among females and the leading cause of cancer death [1]. Hormonal receptor-positive (HR+) breast cancer is the most common molecular subtype of breast cancer and represents about 84% of breast cancer cases [2]. Breast cancer is a heterogenous disease, in which variant molecular features and therapeutic responses were noticed among patients [3]. Epigenetic modifications were amongst the potential players in hormone resistance. De novo and drug induced alterations in DNA methylation, in the promoter regions of genes, have an impact on the initiation and progression of breast cancer [4, 5]. Epigenomic approach through histones acetylation has become a crucial strategy in the way to solve the acquired resistance [6, 7]. The dynamic reaction catalyzed by histone acetyltransferases (HATs) and histone deacetylases (HDACs) has a role in the stimulation or the suppression of tumor growth and progression [4]. Using a combination of HDAC inhibitor with chemotherapy; causes re-sensitization of resistant breast cancer cells to treatment [8, 9]. In addition, the interaction of breast cancer cells with the surrounding microenvironment via interleukins and growth factors was found to have significant impact on the response to endocrine therapy [10]. Immunological regulatory protein such as tumour necrosis factor (TNF), interferon-γ (IFN-γ) and forkhead box P3 (FOXP3) have shown direct/ indirect effects on cancer cell. They mediate the tumor-stromal cell interaction inducing range of matrix metalloproteinases, cytokines and chemokines to promote the tumor development and response to therapy [11, 12].

**Fig. 1:**
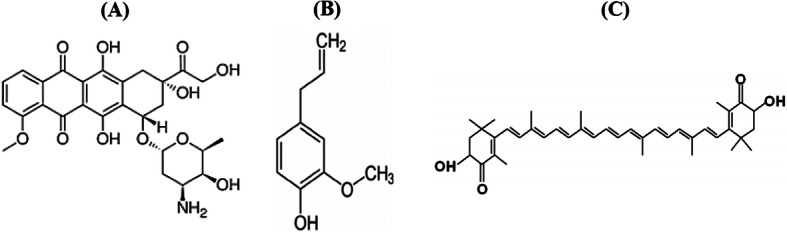
Chemical structure of doxorubicin (**a**), eugenol (**b**) and astaxanthin (**c**)

Doxorubicin (DOX) is an anthracycline antibiotic (Fig. 1a) with broad spectrum anti-tumour activity against many forms of human tumours. DOX induces its antitumor activity via both DNA-single and double strand breaks which is believed to be mediated by DNA intercalation, disruption of topoisomerase-II-mediated DNA repair and generation of free radicals and their damage to cellular membranes, DNA and protein [13]. It is one of the most commonly used chemotherapeutic agents in the treatment of HR+ breast cancer patients with poor prognostic features [14]. Unfortunately, the optimal clinical usefulness of DOX is usually limited secondary to the development of multidrug resistance phenotype as a major limitation observed in HR+ breast cancer treatment [15]. In an attempt to minimize the serious side effects of DOX and to increase its activity, variety of approaches has been investigated using safe and natural compounds [16–18].

Eugenol (EUG) and astaxanthin (AST) are well- known phytochemical which have proven anticancer properties against breast cancer [19–21]. Eugenol (4-allyl (− 2-mthoxyphenol, Fig. 1b) showed versatile pharmacological actions in different types of cancer [22]. It has genoprotective effects against oxidative and methylated DNA damage [23]. Also, it has dose-dependent suppressive and enhancing effects on the immune response in-vitro and in- vivo [24, 25].

Astaxanthin, a marine derived xanthophyll carotenoid (Fig. 1c), has shown to target epigenetic modifying enzymes such as DNA methyltransfersases (DNMTs) and HDACs [26, 27]. Several mechanisms have been proposed for AST induced immunological and anti- inflammatory effects including enhancing both cell-mediated and humoral immune responses. It improves IFN-γ and IL-2 secretion, natural killer cell cytotoxic activity and reduces the intracellular oxidative stress [28, 29]. Accordingly, the current study has been initiated to investigate, on mechanism-based, whether the epigenetic and immunomodulatory effects of EUG and AST could enhance DOX cytotoxicity in HR+ breast cancer cells (MCF7).

## Methods

### Drugs and chemicals

DOX, EUG an AST were obtained from Sigma Aldrich Chemical Co. (St. Louis, MO, USA). Each vial of DOX contains 10 mg doxorubicin hydrochloride in powdered form which was dissolved in DMSO to yield 10 μM then serially diluted in RPMI-1640 medium immediately before use. EUG was obtained in a vial containing 100% pure essential oil. It was dissolved in DMSO and diluted with RPMI-1640 supplemented medium immediately before use. AST was purchased as pink to very dark purple powder stored away from light and dissolved in DMSO to produce stock of 2000 μM. RPMI-1640 Medium, fetal bovine serum, dimethylsulfoxide (DMSO), sodium bicarbonate, Hoechst 3342 solution 1 mg/ml, and rhodamine 123 were all purchased from Sigma Aldrich Chemical Co. (St. Louis, MO, USA). Trichloroacetic acid (TCA) and Triton X-100 were procured from MP Biochemical (Santa Ana, California, USA). All other chemicals and reagents were from standard analytical grade.

### Cells and cell culture

Human breast cancer cell line (MCF7, ATCC® HTB22™) used in this study was obtained from the American Type Culture Collection (Manassas, USA). The adherent cells were grown as monolayer in RPMI- 1640 supplemented with 10% fetal bovine serum, 2 mM L-glutamine, 1.5 g/l sodium bicarbonate and 1% penicillin/streptomycin, and incubated at 37 ͦ C in 5% CO2 atmosphere.

### Methods

### Assessment of cytotoxic activity

Cytotoxicity was determined using sulforhodamine B (SRB) assay as previously described by Skehan et al. [30]. Briefly, exponentially growing cells were seeded in 96-well microtitre plates at an initial density of 5 × 10^3^/well. After 24 h, cells were incubated with different concentration of EUG (0.125–4 mM), AST (5–80 μM), DOX (0.0625–1 μM) alone. Increasing concentrations of DOX were combined with decreasing concentrations of EUG or AST for isobologram combination analysis and synergistic dose selection. Combination of DOX with 1 mM EUG or 40 μM AST were carried out for combination index and fraction affected analyses. For each concentration, three wells were used and incubation was continued for another 48 h. Drug free wells were exposed to vehicles (DMSO 1% v/v) and were used as control. Cells were incubated in a humidified, 5% CO2 atmosphere at 37 ͦ C for 48 h. Cells were fixed with 10% trichloroacetic acid for 1 h at 4 ͦ C and stained with 0.4% SRB for 30 min. The wells were then washed four times with 1% acetic acid, air-dried and the dye was solubilized with 10 mM Tris base (pH 10.5). The optical density (O.D.) was measured spectrophotometrically at 570 nm with the microplate reader (Tecan Sunrise™, Ma¨nnedorf, Switzerland). The experiment was repeated in three independent times. IC_50_ values (the concentration of DOX required to produce 50% inhibition of cell growth) were calculated using sigmoidal dose response curve-fitting models (Graphpad Prizm Software, version 5, GraphPad Software, Inc. Avenida de la Playa La Jolla, USA). Isobologram analysis and combination index calculation was done using CombuSyn software (ComboSyn, Inc., Paramus, NJ., USA).

### Cell cycle and apoptosis analysis with flow cytometry

Control and treated MCF7 cell pellets were stained with DAPI/Triton X-100 staining solution for cell cycle analysis and with propidium iodide for apoptosis analysis [31]. A flow cytometer (Becton and Dickinson San Jose, CA., USA) equipped with electronic doublet-discrimination capability was used to detect stained nuclei and emitted fluorescent light primarily at wavelengths between 580 and 650 nm. The FACscan fluroscence 2 (FL2) detector equipped with a 585/42 band pass filter was used to analyze light emitted between 564 and 606 nm.

### RNA extraction, cDNA synthesis and real time PCR

Total RNA was extracted from control and treated cell pellets with total RNA purification kit (Direct-Zol RNA Kit, Zymo Research, Germany). cDNA synthesis was performed using Revert Aid First Strand cDNA synthesis kit (ThermoFisher, UK), in which 1 μl reverse transcriptase enzyme was added to 10 μl RNA sample in the presence of 2 μl of RT buffer, 0.8 μl dNTP mix, 2 μl random primers, 1 μl RNase inhibitor and 3.2 μl nuclease-free water. The cycling conditions were 25 °C for 10 min, 37 °C for 120 min and 85 °C for 60 min. Quantitative real time PCR was conducted by Applied Biosystems syber green PCR master mix (USA). 1 μl of cDNA was added to 25 μl master mixtures of CXR Reference Dye, forward and reverse primers and double distilled H_2_O. Initial denaturation at 95 °C for 10 min, followed by 40 cycles of denaturing at 95 °C for 15 s, and annealing at 62 °C for 1 min was performed for all analyses in triplicate on a 7500 Real-Time PCR System (Applied Biosystems, Foster City, CA, USA). The cycle threshold (Ct) was determined automatically. Three samples without a template were always included as a no template control. Reverse and forward sequences of primers genes encoding for mRNA transcript of TNFα, IFNγ, FOXP3, BAX, BCl2 and caspase 8 genes were designed by NCBI- NIH tool and the sequences were summarized in Table 1. Fold change of genes expression (2^-∆∆Ct^) was calculated after normalization to housekeeping gene (β-actin) and genes expression in control samples.

**Table 1:** Primers for qRT- PCR

Gene	Forward Primer	Reverse Primer
***TNFα***	CTGAACTTCGGGGTGATCG	GCTTGGTGGTTTGCTACGAC
***IFNγ***	ACTGTCGCCAGCAGCTAAAA	TATTGCAGGCAGGACAACCA
***FOXP3***	CCCAGGAAAGACAGCAACCTT	TTCTCACAACCAGGCCACTTG
***BAX***	GCCCTTTTGCTTCAGGGTTT	TCCAATGTCCAGCCTTTG
***BCl2***	CGGAGGCTGGGATGCCTTTG	TTTGGGGCAGGCATGTTGAC
***caspase 8***	TTCTCCCTACAGGGTCATGC	GCAGGCTCAAGTCATCTTCC
***β- actin***	CCAGAGCAAGAGAGGTATCC	CTGTGGTGGTGAAGCTGTAG

### SDS-polyacrylamide gel electrophoresis and immunoblot analysis

Control and treated cells were harvested, washed twice with ice-cold phosphate buffered saline, centifuged and pelleted at 1200 r/min for 5 min. The cell pellets were then lysed in lysis buffer containing 150 mM sodium chloride, 10 mM Tris, 0.2% Triton X-100, 0.3% NP-40, 0.2 mM sodium vanadiumoxide and protease inhibitor cocktail, pH 7.7. The supernatants were collected after centrifugation at 14,000 r/min for 15 min at 4 ͦ C, and the protein content was determined by the Bradford method [32]. Aliquots of protein supernatants containing equal amounts of protein and sodium dodecyl sulphate (SDS)-reducing buffer were boiled for 5 min, electrophoresed on SDS-polyacrylamide gels and transferred to polyvinylidene difluoride membranes. The membranes were blocked with 5% non-fat drymilk and probed with specific primary antibodies of monoclonal antihuman aromatase (Biospes, Aachen, Germany), EGFR (Sigma Aldrich, USA), CK7 (Bioss, Boston, USA) and LC3B (Invitrogen, USA) antibodies followed by incubation with peroxidase-conjugated secondary antibodies. The blots were developed with Amersham ECL Western Blotting Detection Reagents (GE Healthcare, Amersham Place, Little Chalfont, UK) according to themanufacturer’s protocol. The blots were quantified by Scion image software (Scion Corporation, version 0.4.0.3, Maryland, USA) and protein loading was corrected for β-actin as loading control.

### Histones extraction and the determination of global H3 and H4 acetylation

Cell pellets were suspended in triton extraction buffer (0.5% Triton in phosphate buffered saline, 2 mM phenylmethylsulfonyl fluoride and 0.02% NaN3), and lysed on ice for 10 min with gentle stirring. After centrifugation, cell lysate was transferred to a new vial and the residual cells were resuspended in the extraction buffer (0.5 N HCl + 10% glycerol) and incubated on ice for 30 min. The supernatant fraction was taken to a new vial and 8 volumes of acetone was added and left at − 20 °C overnight. The Protein concentration was quantified in the remaining dry pellet by Coomassie protein assay kit following Bradford method [32]. The EpiQuik™ Total Histone H3 and H4 Acetylation Detection Fast Kits (Epigentek, Farmingdale, NY, USA) were used according to the manufacturer` protocol. The global content of acetylated histones in treated samples was calculated from the protein calibration curve in ng/ total histones protein, and then the % of histones acetylation in was calculated normalized to the level of acetylated histones in untreated control.

### Enzyme-linked immunosorbent assay for caspase 3

The concentration of executioner caspase 3 active subunit was measured in the lysate of MCF-7 cells using the Quantikine human active caspase-3 immunoassay kit (R&D system, Minneapolis, MN, USA). The amount of caspase-3 was calculated from a standard curve, and the results are presented as relative % of active caspase-3 to untreated control.

### Determination of the activity of multidrug resistance (MDR) via rhodamine-123 and Hoechst dyes

Rhodamine-123 and Hoechst 3342 dyes are substrates for MDR genes and the proteins codified by these genes including p-glycoprotein (P-gp), MDR associated protein, breast cancer resistant protein and lung-resistant related protein [33]. Accumulation of Rhodamine-123 and Hoechst dyes in the cells is inversely related to MDR activity [34]. In brief, adherent control and treated cells were incubated with 5.25 μM of Rhodamine 123 and 5 μg/ml of Hoechst 33342 dye for 30 min at 37 ͦ C in a 5% CO2 incubator. After incubation, cells were washed, scrapper collected, re-suspended and physically lysed in distilled water for immediate fluorescence analysis. Cellular uptake of Rhodamine 123 was detected at excitation 485 nm and emission 535 nm, while cellular uptake of Hoechst 3342 was detected at excitation 360 nm and emission 450 nm using fluorescence spectroscopy (Kontron SFM25, Tresser Instruments, Rossdorf, Germany).

### Statistical analysis

All data are expressed as mean ± SD of three separate experiments, each performed in triplicates. Differences between groups were tested for statistical significance using one-way analysis of variance (ANOVA) followed by Dunnette for comparing all means with control in the SRB cytotoxicity study and Tukey-kramer for multiple comparison in rest of the experiments. A student t-test was used for comparison between the mean in DOX alone and the corresponding mean in DOX combined with either EUG or AST in SRB cytotoxicity study. Nonparametric ANOVA was carried out for comparison between three blots of Western blotting using the Kruskal–Willis test. The 0.05 level of probability was used as the criterion of significance using GraphPad InStat, version 4.0 (GraphPad, San Diego, California, USA).

## Results

### Effect of EUG or AST on DOX cytotoxicity in MCF-7 cells

**Fig. 2:**
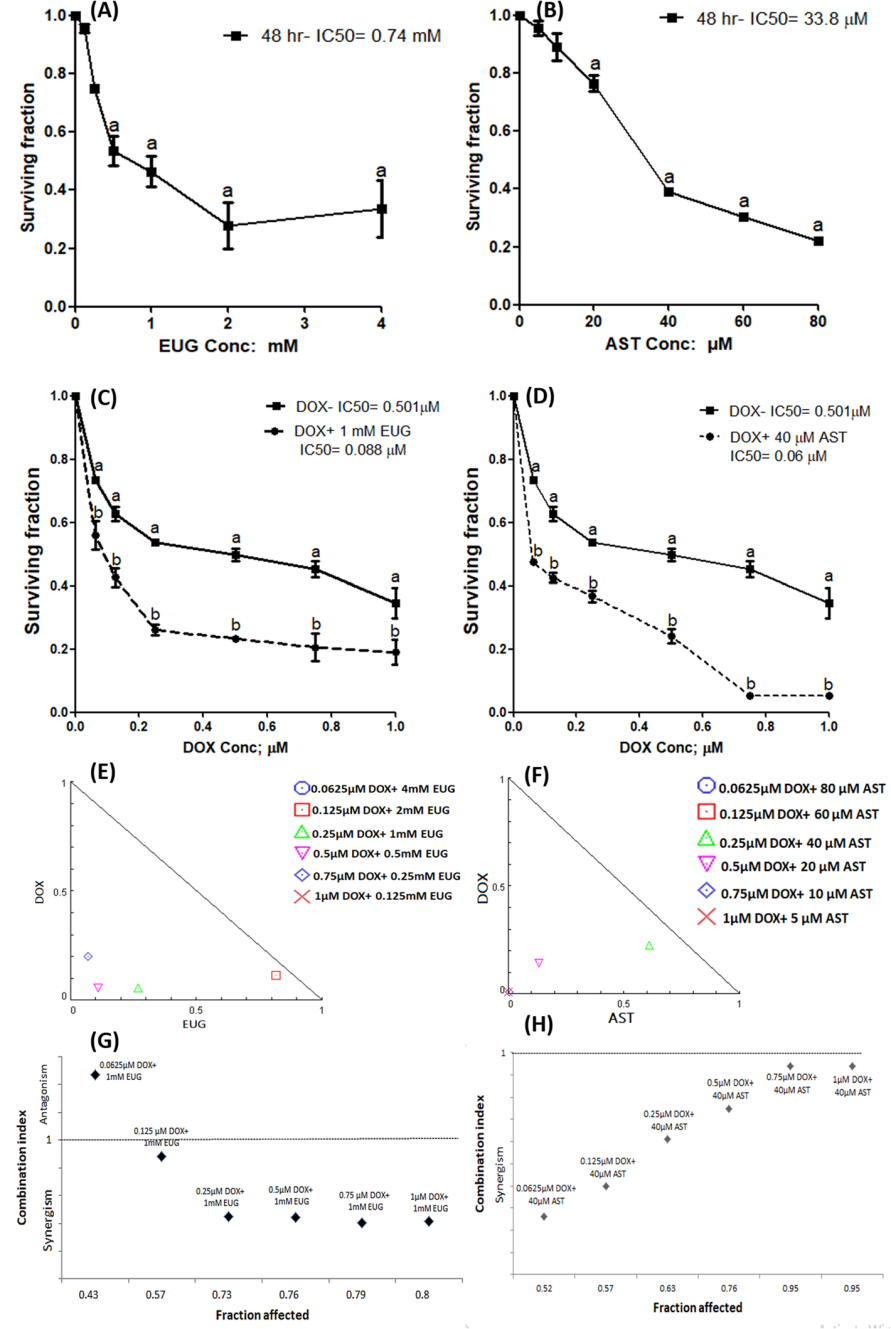
Effect of EUG (**a**), AST (**b**), DOX plus EUG (**c**) and DOX plus AST (**d**) on survival of MCF7 cells. Normalized isobologram constructed for the combination of increasing concentrations of DOX with decreasing concentrations of EUG (**e**) and AST (**f**). The combination index produced from 1 mM EUG combined with 0.25 μM DOX (**g**) and 40 μM AST combined with 0.25 μM DOX (**h**). Data are expressed as mean ± SD of three separate experiments, each performed in triplicates. ^a^ indicates significant change from control at *P* ≤ 0.05 using one-way ANOVA followed by Dunette as a post ANOVA test and ^b^ indicates significant from DOX alone at P ≤ 0.05 using student t-test.

Figure 2 shows the effects of EUG (A) and AST (B) on the survival of MCF7 cells after 48 h incubation period. EUG and AST caused a concentration-dependent cell death. The IC50 recorded for EUG was 0.74 mM, and for AST was 33.8 μM. The concentration that produced significant decrease of survival in MCF7 for EUG and AST was the nearest concentration above the IC_50_ which found to be 1 mM of EUG and 40 μM of AST. Fig. 2c and d showed the effects of 1 mM EUG (C) and 40 μM AST (D**)** combined with various concentrations (0.0625–1 μM) of DOX for 48 h on the survival of MCF7 cells. DOX alone resulted in concentration-dependent cytotoxicity with IC_50_ of 0.5 μM. Both EUG and AST significantly increased DOX cytotoxicity manifested as a significant decrease in DOX IC_50_ from 0.5 μM to 0.088 with EUG **(C)** and to 0.06 μM with AST (D).

Normalized isobologram was constructed for various descending concentrations of EUG or AST with various ascending concentrations of DOX as shown in (Fig. 2e and f). EUG showed synergistic cytotoxic effect with DOX in combinations: 0.125 μM DOX+ 2 mM EUG, 0.25 μM DOX+ 1 mM EUG, 0.5 μM DOX+ 0.5 mM EUG and 0.75 μM DOX+ 0.25 mM EUG (Fig. 2e), while AST showed synergistic cytotoxic effect with DOX in combinations: 0.25 μM DOX+ 40 μM AST and 0.5 μM DOX+ 20 μM AST (Fig. 2f). The combination index-fraction affected graph was drawn and presented in (Fig. 2g and h) for EUG and AST, respectively. The combination of 0.25 μM DOX+ 1 mM EUG caused reduction in the cell growth of MCF7 by a fraction of 0.73, and a synergistic combination index of 0.44 (Fig. 2g). The combination of 0.25 μM DOX+ 40 μM AST caused reduction in the cell growth of MCF7 by a fraction of 0.61, and a synergistic combination index of 0.63 (Fig. 2h). EUG and AST showed synergistic cytotoxic effects upon combination with DOX against the growth of MCF-7 cells.

### The effect of EUG and AST on the level of histones acetylation in DOX treated MCF7 cells

**Fig. 3:**
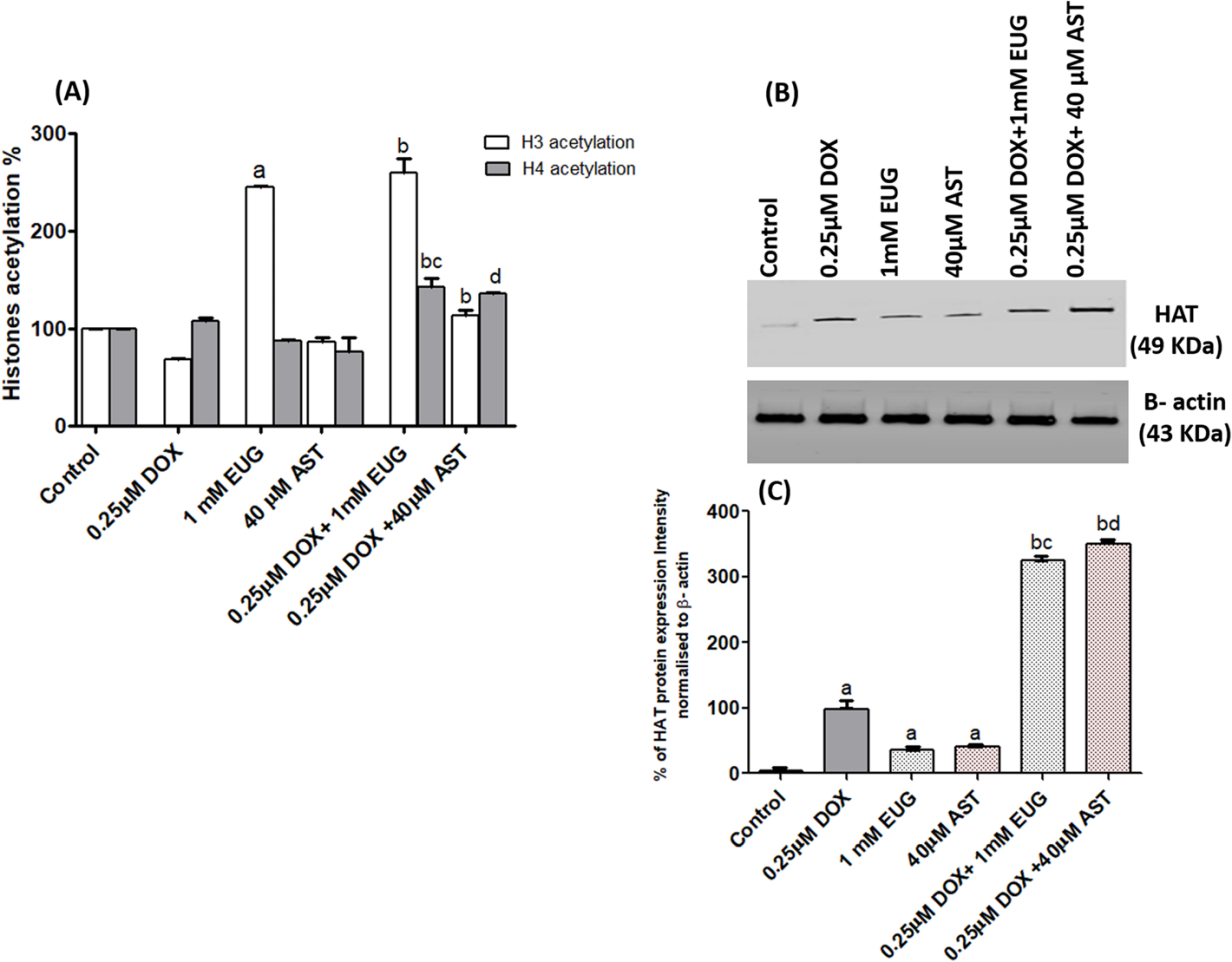
Effect of DOX, EUG, AST and their combination on Histones (H3 and H4) acetylation % (**a**), HAT protein expression (**b**), and % of HAT protein intensity normalised to β-actin (**c**). Data are expressed as mean ± SD of three separate experiments, each performed in triplicates. ^a,b,c and d^ indicate significant from control, DOX, EUG and AST, respectively at P ≤ 0.05 using one-way ANOVA followed by Tukey-Kramer as a post ANOVA test.

Significant increase in H3 acetylation was shown only with EUG treatment compared with control (Fig. 3a). The combination of DOX with EUG caused a significant increase in both H3 and H4 histones acetylation, while with AST it caused only a significant increase in H3 histone acetylation, compared with DOX alone (Fig. 3a). Significant increase in H4 histone acetylation was demonstrated in both EUG and AST combination compared with their corresponding single treatment of each (Fig. 3a). HAT protein expression was significantly increased in DOX, EUG and AST treated cells compared with control (Fig. 3b and c). Also, a significant overexpression of HAT was demonstrated in cells treated with DOX combined with EUG and AST compared with DOX, EUG and AST each alone (Fig 3 and c). EUG and AST showed an epigenetic potential through increasing global histones acetylation and HAT protein expression.

### Immunological modulation of EUG and AST to DOX activity on FOXP3, IFNγ, TNFα, aromatase and EGFR expression in MCF7 cells

**Fig. 4:**
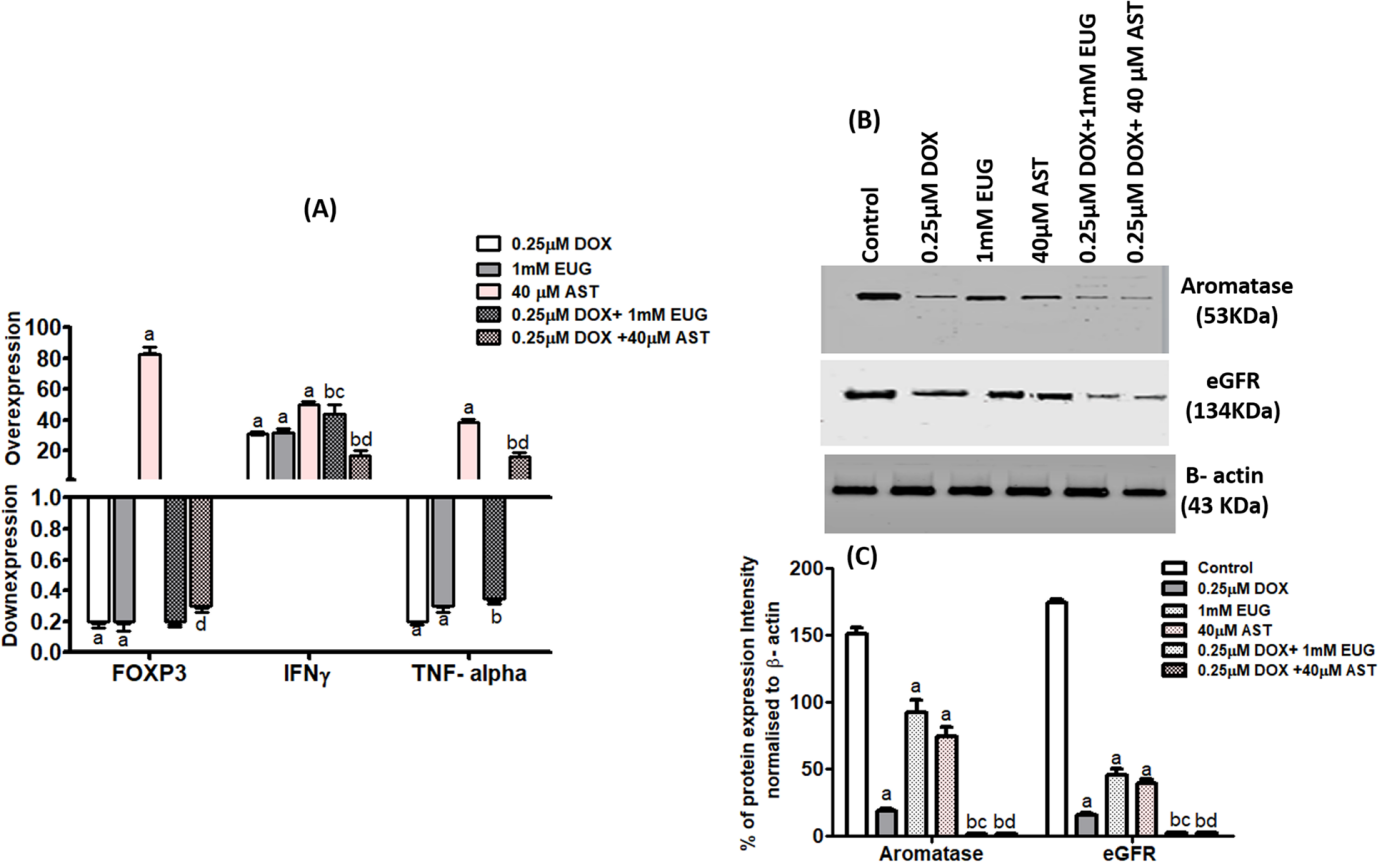
Effect of DOX, EUG, AST and their combination on FOXP3, IFNγ and TNFα mRNA expression (**a**), aromatase and eGFR protein expression (), and % of aromatase and eGFR protein intensity normalised to β- actin (**c**). Data are expressed as mean ± SD of three separate experiments, each performed in triplicates. ^a,b,c and d^ indicate significant from control, DOX, EUG and AST, respectively at P ≤ 0.05 using one-way ANOVA followed by Tukey-Kramer as a post ANOVA test.

In (Fig. 4a), single treatment with DOX and EUG caused a significant decrease in FOXP3 and TNFα, but increased mRNA expression of IFNγ compared with control. Single treatment with AST caused significant mRNA overexpression of FOXP3, IFNγ and TNFα compared with control. EUG combination caused a significant increase in IFNγ but it decreased TNFα compared with DOX. AST plus DOX combination caused a significant decrease in FOXP3 expression compared with AST alone and also caused a significant decrease in IFNγ expression compared with single DOX and AST. The expression of TNFα in AST plus DOX was higher than in DOX but lower than in AST alone. In (Fig. 4b and c), single treatment with DOX, EUG and AST caused significant decrease in aromatase and EGFR protein expression compared with control. DOX combinations with EUG and AST caused further decrease in aromatase and EGFR protein expression compared with single treatment with DOX, EUG and AST. EUG and AST decreased protein expression of aromatase and EGFR.

### EUG and AST modulated the multidrug resistance of MCF7 cells to DOX

**Fig. 5:**
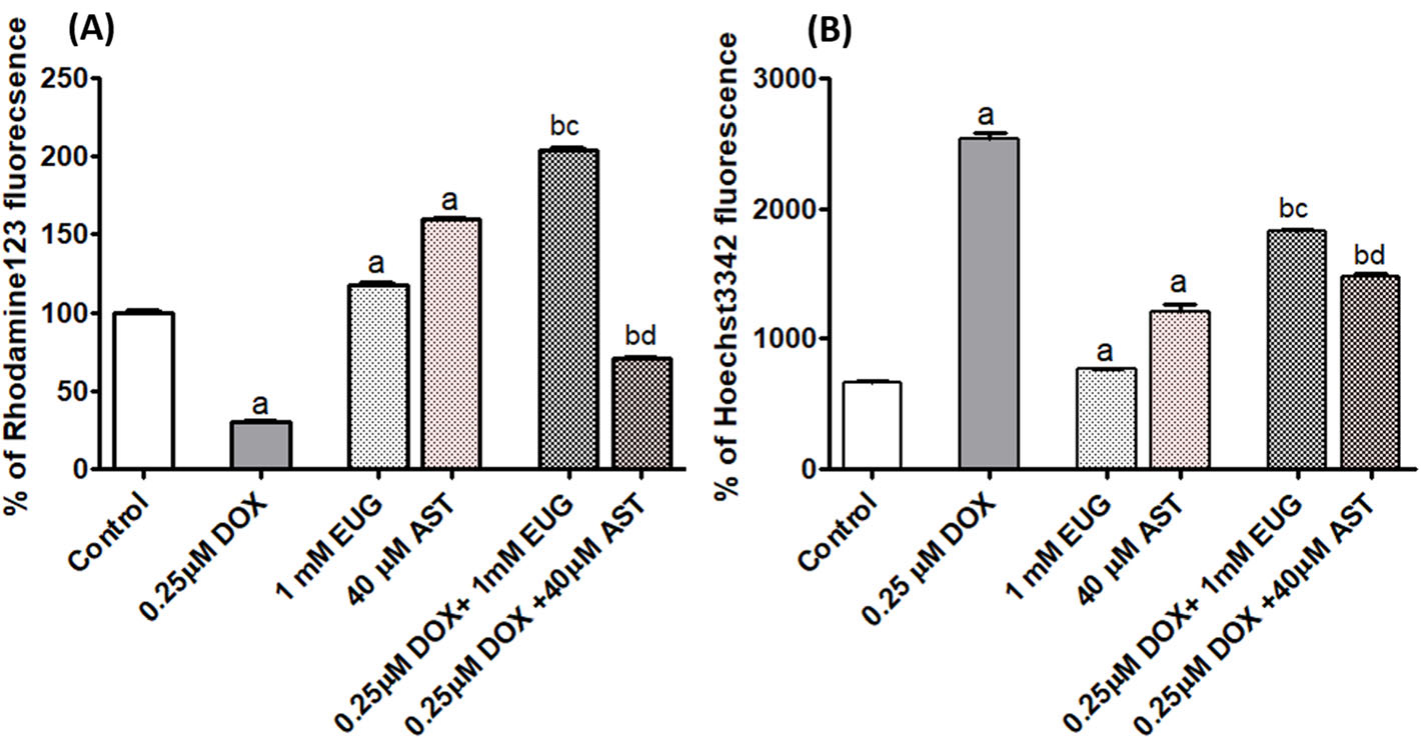
Effect of DOX, EUG, AST and their combination on % of rhodamine123 uptake (**a**) and % of hoechst3342 uptake (**b**). Data are expressed as mean ± SD of three separate experiments, each performed in triplicates. ^a,b, c and d^ indicate significant from control, DOX, EUG and AST, respectively at P ≤ 0.05 using one-way ANOVA followed by Tukey-Kramer as a post ANOVA test.

DOX treated cells had significant lower uptake of rhodamine 123 (Fig. 5a), but higher uptake of hoechst3342 (Fig. 5b), compared with control. EUG and AST treated cells had significant higher uptake of rhodamine 123 (Fig. 5a), and hoechst3342 (Fig. 5b), compared with control. The combination of DOX with EUG resulted in significant higher uptake of rhodamine123 compared with single treatment with DOX and EUG (Fig. 5a). The combination of DOX with AST resulted in significant higher uptake of rhodamine123 compared with single treatment with DOX but lower uptake of rhodamine123 compared with single treatment with AST (Fig. 5a). Cells treated with EUG and AST combination showed lower Hoechst 3342 uptake than cells treated with DOX, but higher Hoechst 3342 uptake than cells treated with single EUG or AST (Fig. 5b). EUG and AST decreased MDR activity.

### Cell cycle and apoptosis analysis with flow cytometry

**Fig. 6:**
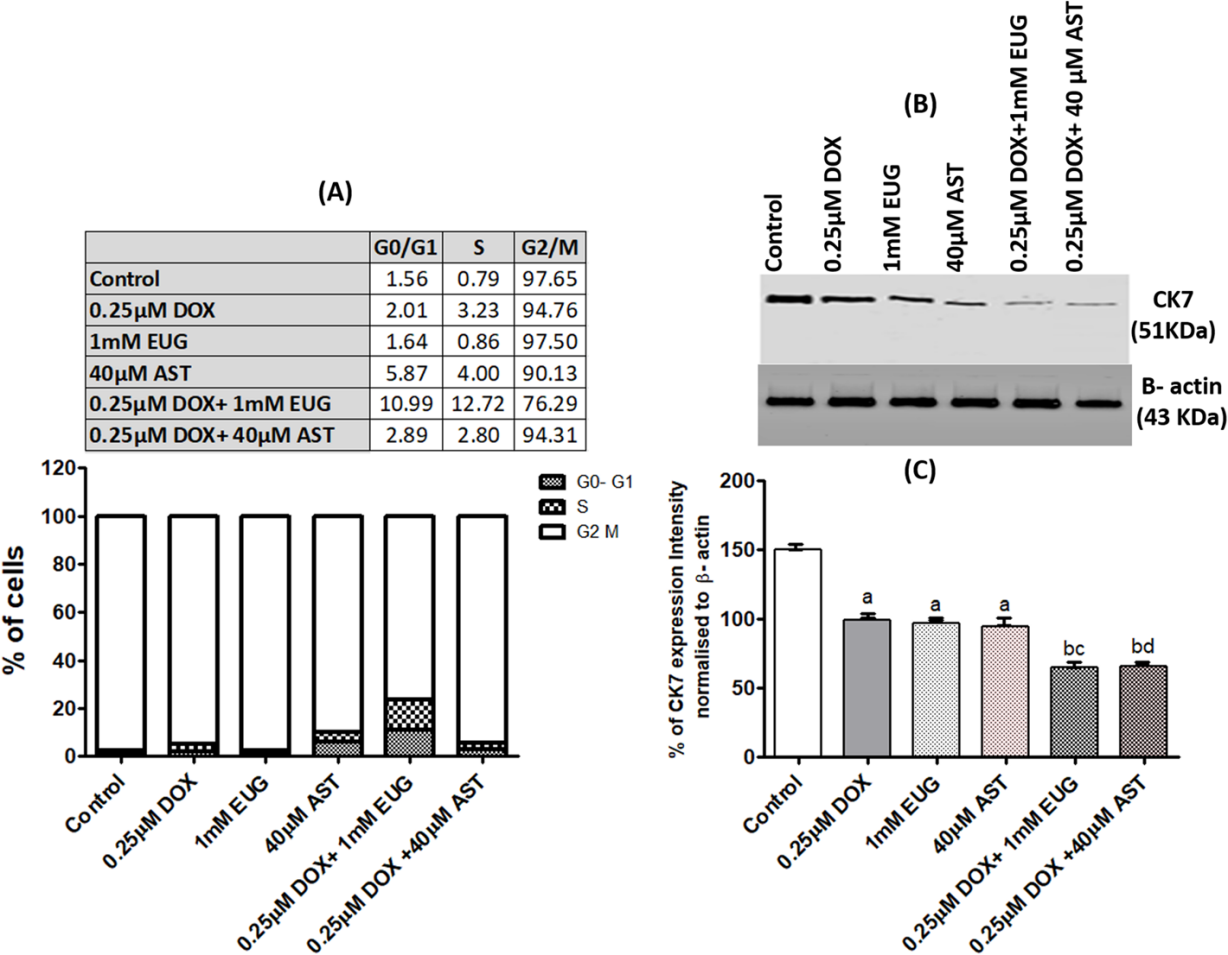
Effect of DOX, EUG, AST and their combination on % of cells at the phases of cell cycle (**a**), CK7 protein expression (**b**), and % of CK7 protein intensity normalised to β-actin (**c**). Data are expressed as mean ± SD of three separate experiments, each performed in triplicates. ^a,b, c and d^ indicate significant from control, DOX, EUG and AST, respectively at P ≤ 0.05 using one-way ANOVA followed by Tukey-Kramer as a post ANOVA test.

**Fig. 7:**
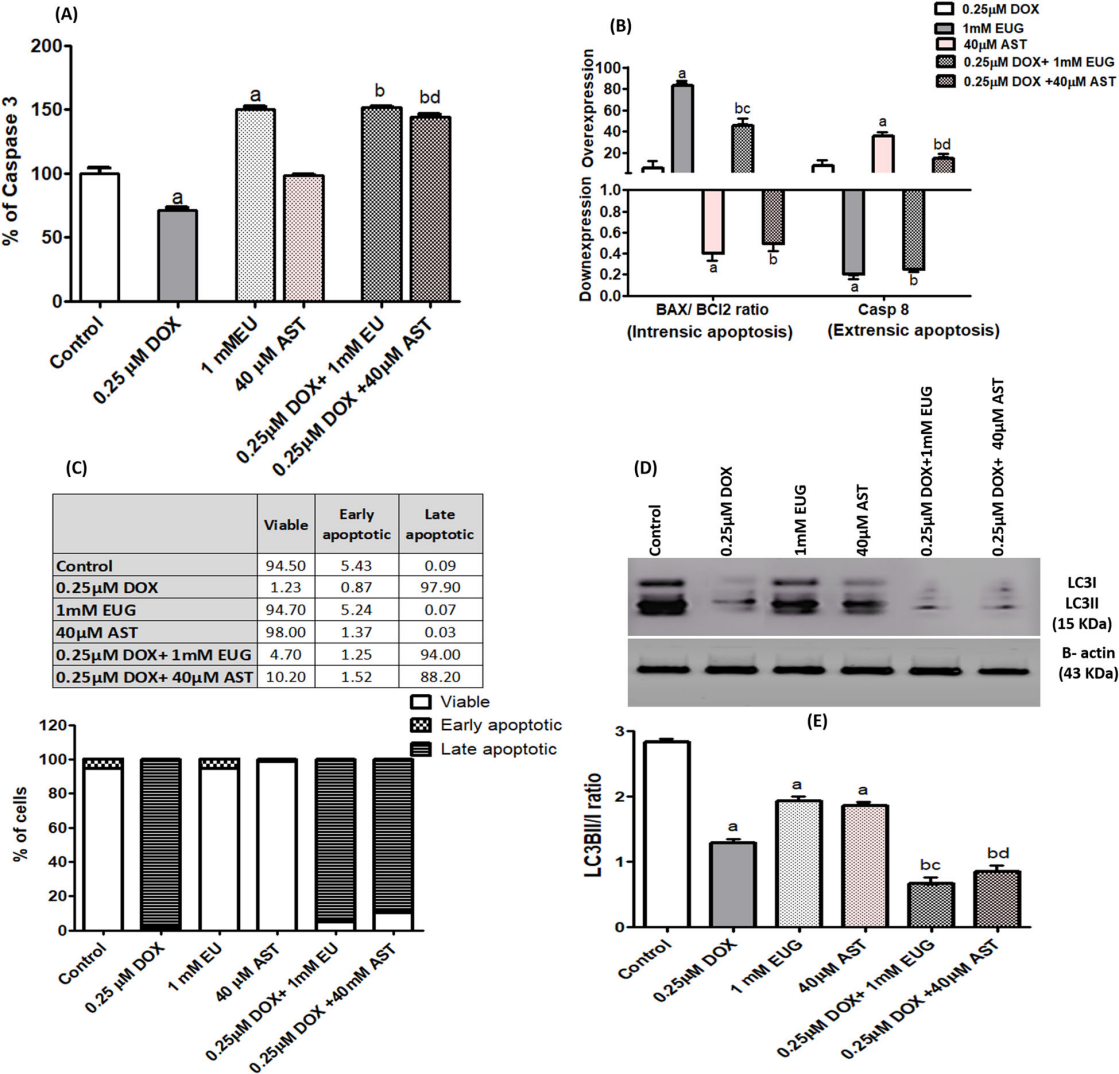
Effect of DOX, EUG, AST and their combination on % of caspase 3 (**a**), mRNA expression of BAX, BCl2 and caspase 8 (**b**), % of cells according to propidium iodide staining (**c**), LC3I and LC3II protein expression (**d**), and LC3B II/I ratio (**e**). Data are expressed as mean ± SD of three separate experiments, each performed in triplicates. ^a,b, c and d^ indicate significant from control, DOX, EUG and AST, respectively at P ≤ 0.05 using one-way ANOVA followed by Tukey-Kramer as a post ANOVA test.

Increased accumulation of cells in G0/G1 was observed after treatment with DOX (2.01%) and AST (5.87%) each alone, whereas EUG alone showed accumulation (1.64%) similar to control (1.56%). Fascinatingly, the combination of DOX with EUG resulted in five folds increase in the percentage of cells in G0/G1 (10.99%) compared with cells treated with DOX alone (2.01%). Although AST alone showed the highest accumulation of cells in the G0/G1 (5.87%), it has no effect on DOX- induced accumulation of cells in G0/G1 (Fig. 6a). EUG and AST combination caused early apoptosis to 1.25% of cells compared with 0.87% of cells treated with DOX alone (Fig. 7c). EUG and AST caused early apoptosis to DOX treated MCF7 cells.

### Both EUG and AST combinations inhibited the expression of CK7

Protein expression of luminal differentiation marker (cytokeratin 7: CK7) was shown to be significantly reduced in MCF7 cells treated with single DOX, EUG and AST compared with control, and further reduction was observed when DOX combined with EUG and AST compared with single DOX, EUG and AST (Fig. 6b). EUG and AST decreased CK7 protein expression.

### EUG induced intrinsic apoptosis through BAX/ BCl2 while AST induced extrinsic apoptosis through caspase 8 and both activated caspase 3 but reduced LC3B expression

The percentage of active caspase 3 subunit was shown to be significantly reduced in DOX treated cells, while significantly induced in EUG treated cells, compared with control. Combination of EUG with DOX significantly induced capase 3% compared with DOX alone. While combination of AST with DOX significantly induced capase 3% compared with DOX and AST alone (Fig. 7a). Single treatment with EUG showed significant overexpression of BAX/ BCl2 ratio, but downexpression of caspase 8, compared with control. On the contrary, AST showed significant downexpression of BAX/ BCl2 ratio, but overexpression of caspase 8, compared with control. Cells treated with EUG combination revealed significant higher level of BAX/ BCl2 ratio but lower level caspase 8 compared with cell treated with DOX alone. Cells treated with EUG combination revealed significant lower level of BAX/ BCl2 ratio when compared with cells treated with EUG alone. When AST combination compared with DOX, significant lower level of BAX/ BCl2 ratio but significant higher level of caspase 8 was revealed. The level of caspase 8 mRNA expression in cells treated with AST combination was lower than in cells treated with AST alone (Fig. 7b). The protein expression of autophagic marker (LC3BII/I ratio) was shown to be significantly reduced in MCF7 cells treated with single DOX, EUG and AST compared with control, and further reduction was observed when EUG and AST combinations compared with single DOX, EUG and AST (Fig. 7d). EUG induced apoptosis by increasing BAX/ BCl2 ratio, while AST induced apoptosis via increasing caspase-8 expression.

## Discussion

DOX is among the most commonly used anticancer drugs with broad spectrum antitumor activity against several human tumours including breast cancer. Unfortunately, the optimal clinical benefits of DOX are limited secondary to its detrimental cardiomyopathy and the development of MDR [35, 36]. Earlier studies have demonstrated that EUG attenuated DOX-induced genotoxicity and cardiotoxicity [16, 17]. Similarly, AST treatment significantly protected against DOX-induced oxidative and inflammatory insults and downregulated the overactive apoptotic machineries [18]. Therefore, the current study extended such beneficial effects of EUG and AST by testing their combination with DOX in MCF7 to investigate, on mechanism-based, whether EUG and AST could enhance the sensitivity of HR+ MCF7 cells to DOX, and if so, whether these effects are linked to MDR-P-gp pathway and/or the non-MDR-epigenetic immunomodulation.

Data presented in the current study showed that EUG and AST induced remarkable enhancement in DOX cytotoxicity in MCF-7 cells manifested as a significant 82 and 88% decrease in the IC_50_, respectively as compared to DOX alone (Fig. 2). Our results confirmed earlier study which has reported that EUG induced cytotoxic activity against different molecular subtypes of breast cancer cells and induced apoptosis in a p53-independent manner [19]. Using MCF7 cells, Vidhya and Devaraj [37] reported that EUG treatment caused concentration and time-dependent inhibition of growth and proliferation of the cells and increased the percentage of apoptotic cells and DNA fragmentation through depleting the level of intracellular glutathione and increasing the level of lipid peroxidation.

The current results showed that addition of EUG to DOX-treated cells resulted in shifting MCF-7 cells toward the S and G0/ G1 phases and induced the intrinsic apoptosis through the higher BAX to BCl2 ratio (Fig. 7b). In concordance, DOX induced mitochondrial-dependent apoptosis by down-regulation of Bcl-xL and up-regulation of Bax and caspase-9 expressions in MCF7 cells [38]. In addition, Júnior et al. [39] pointed that EUG induced apoptosis in cancer cells through promoting the production of reactive oxygen species and reducing the mitochondria potential through the upregulation of Bax expression causing the abrogation of cells from the G2/M of phase of cell-cycle. Moreover, the chemo-sensitivity of MCF-7 cells to EUG was found to be mainly mediated through the distortion of mitochondrial membrane integrity with the consequent release of cytochrome-c and lactate dehydrogenase into culture media at EUG concentration more than its effective concentration 50 [20]. Therefore, one can anticipate a synergistic harmony between DOX and EUG in the molecular insights leading to apoptosis. On the other hand, our results revealed that DOX combination with AST induced apoptosis through overexpression of caspase 8, the key enzyme of the extrinsic apoptosis [40], and there was insignificant change in the distribution of cells through the phases of cell cycle. Under similar experimental condition, Sharifi et al. reported that DOX induced non-significant change in the level of caspase 8 after 24 h incubation, but significantly increased its level after 48 and 72 h of incubation [38]. Different modes of AST driving apoptosis when combined with DOX were recently reported [41, 42]. It has been reported that, the pro-oxidant property of AST was the main force for selective apoptosis MCF7 cells in which growth inhibition was increased in a synergistic pattern rather than normal breast epithelial cells (MCF-10A) [41]. In vivo study concluded that AST caused up-regulation of tumor suppressor p53 gene, potentiating DOX cytotoxicity and apoptosis against mammary tumor cells but accumulating them in the G2/M phase of the cell cycle [42].

Earlier studies confirmed that DOX-treated MCF-7 cell developed varying degrees of resistance depending on the concentration of DOX used [43] and that DOX is a selective P-gp substrate, and induced expression of MDR in tumor cells [44]. Therefore, to test EUG and AST sensitization of MCF7 to DOX treatment, the current study followed two approaches; one through the direct measurement of MDR-Pgp post-translational activity, and the other is non-MDR mediated through the measurement of CK7, LC3B, immunological and epigenetic markers. In this study, the sensitization of MCF7 cells to DOX treatment was investigated by the functional assay of MDR depending on the intracellular accumulation of Rhodamine 123 and Hoechst dyes [45, 46]. Our results showed that treatment with DOX alone caused reduced intracellular Rhodamine 123 accumulation but induced that of Hoeacsht 3342, indicating the basal sensitive nature of our MCF7 cells (Fig. 5b). Combined use of EUG or AST with DOX induced higher uptake of Rhodamine123, but lower of Hoechst 3342 than DOX alone, indicating the switch on induction of the P-gp activity upon combination treatment. In DOX resistant breast cancer cells, the pumping of DOX out of cells was shown to be dependent on ATP avaliability [47]. The possibility of alterations in P-gp activity might be explained by changes in P-gp protein levels in DOX treated cells [33]. Moreover, P-gp activity may be modulated by cellular components such as membrane proteins, membrane-anchoring proteins or membrane-lipid composition [48]. Alterations of mitochondrial membrane potential and intracellular ATP level by EUG [20, 49] and AST were reported [50, 51] and explained the difference in the intracellular accumulation of DOX in combination than single agent. By reversing DOX resistance, DOX accumulate in MCF7 cells by endocytosis bypassing the effect of P-gp mediated efflux [52].

In our study, the combination of DOX with both EUG and AST significantly decreased CK7 expression. It is well known that cells with positive expression of CK7 exhibited resistance to DOX treatment [53, 54]. The immunomodulation potency of EUG and AST on the differentiation of mesenchymal stem cells which affect therapy applications was reported [55, 56]. For further elucidation of the non-MDR directed EUG and AST sensitization to DOX anticancer activity in MCF7 cells, we investigated the effect of DOX alone and combined with EUG or AST on EGFR and aromatase expression. Our results showed that EUG and AST alone induced mild anti-EGFR effect. However, after combined treatment with DOX, EUG or AST, caused marked reduction in EGFR and aromatase proteins expression. Our results are consistent with previous studies which reported that increased the number of EGFR in tumor was associated with DOX resistance [57], and that combination of DOX with anti-EGFR therapy enhanced DOX effects against EGFR overexpressed tumor xenografts [58]. The ability of EUG to block HER2/PI3K-AKT signalling in breast precancerous lesions was reported [59]. In cervical cancer cells, AST reduced the expression of EGFR and interfere with EGF binding, thereby inducing apoptosis [60]. Aromatase and estrogen receptor α (ER α) are two key proteins which are responsible for the proliferation of MCF7 cells [61]. Our results showed a significant inhibition in aromatase protein expression after treatment with DOX. Pritchard et al. showed that DOX induced changes on estrogen signaling relevant to its therapeutic efficacy [14]. However, the presence of physiological estrogen levels will reverse DOX cytotoxic effect in breast cancer cells [14]. The anti-oxidant and anti-inflammatory activities of EUG and AST were suggested to contribute in their anti-aromatase effects. The idea was exported from the well- known aromatase inhibitor (exemestane) which showed non-estrogenic chemopreventive activity through its anti-inflammatory and reactive oxygen species scavenging properties [62]. Red clover flowers (from which EUG was extracted) were found to inhibit aromatase at low concentrations, while they had estrogenic activity at high concentrations [63]. Moreover, we suggest that the known anti-hyperlipidemic effect of AST [64] may also contribute in its aromatase inhibitory activity [65]. Especially there is a classical correlation between ER-positive breast cancers and adipose tissue expression of aromatase, which is considered a local source of estrogens [66].

Autophagy is the main reason for acquired resistance phenotype in ER+ breast cancer, and its molecular target LC3B is found to be highly expressed in the breast cancer tissues [67]. Data presented in the current study showed that the expression of LC3BI and II was vanished in cells treated with DOX combined with EUG or AST as compared to each alone (Fig. 7d). In colorectal cancer cells, EUG was identified as pro-autophagic compound [68], where the active fraction of clove (oleanonic acid and eugenol) increased LC3B I and II and Beclin-1 protein expression. AST modulates the signaling pathways that regulate autophagy [69], either by stimulation as shown in an experimental model of non-alcoholic fatty liver disease [70], or by inhibition as shown in the pancreas by inhibiting the JAK/STAT3 pathway [71]. Also the anti-oxidant and reactive oxygen scavengining activities of EUG [72] and AST [73] during DOX treatment may explain for the classical autophagic inhibition usually observed in that context with other anti-oxidant compounds [74].

Under our experimental condition; the epigenetic potential of EUG and AST was evaluated, and it was found that EUG alone and the combinations of DOX with 1 mM EUG or 40 μM AST significantly induced the level of global histones acetylation along with increasing the protein expression of histone acetyl transferase (Figs. 3a, b and c). This histone deacetylase inhibition activity observed with EUG illustrates its proautophagic effect and intrinsic apoptotic cell death. On the same way, most of histone deacetylase inhibitors can induce mitochondria-mediated apoptosis and provoke autophagy-induced caspase-independent cell death [75, 76]. The inhibitory effect of AST on histone deacetylase 9 expressions was observed [77]. Combination of DOX with histone deacetylase inhibitors promoted DOX-induced apoptosis through a mechanism that involved induction of tumor suppressor gene PTEN which is the major negative regulator of the PI3K/Akt cellular survival pathway [78]. According to that, the proven induction of H3 and H4 histone acetylation by EUG and AST was suggested to contribute in the DOX synergistic cell death observed in this study.

In the present study, exposure of MCF7 cells to either DOX or EUG alone significantly decreased expression of FOXP3 and TNFα and increased expression of IFNγ, while AST caused overexpression of the three genes (Fig. 4a). DOX combined with EUG significantly increased IFNγ expression and decreased TNFα expression when compared with DOX alone. The vice versa was observed when AST combined with DOX where a significant reduction in IFNγ expression accompanied with TNFα overexpression in comparison with DOX alone. Both EUG and AST combinations showed no change in FOXP3 expression compared with single treatment with DOX. Moreover, DOX induced a remarkable increase in Foxp3 protein in MCF7 cells that was associated with the phosphorylation of p53 [79]. Recent study suggested that the antitumor effect of EUG was secondary to its regulatory action on the production of inflammatory mediators from macrophages Barboza et al. [25]. EUG reduced TNF-*α* and IL-1*β* as well as the NF-*κ*B, ERK1/2, and p38 MAPK signaling pathways [80]. EUG exhibited synergistic effect when combined with gemcitabine by downregulating the expression of Bcl-2, COX-2 and IL1-β [81]. It has been also reported that EUG induced downregulation of TNF-*α* in LPS-activated macrophages, which was associated with antigenotoxic activity when DNA damage was induced with DOX [16]. Additionally, it was reported that EUG synergistically increased cisplatin cytotoxicity against triple negative breast cancer through the inhibition of NF-*κ*B signalling pathway, p50 and p65 subunits phosphorylation, and IL-6 and IL-8 downregulation [82]. In tumor environment, it has been reported that AST decreased the amount of inflammatory markers such as TNF-α, IL-6, and IFNγ via NF*k*-B inhibition [83]. In mouse breast cancer model, AST treatment caused higher levels of apoptotic cancer cells and [84], promoted early check and elimination of cells undergoing malignant transformation by activating immune surveillance [85] and prevented cancer cell growth in cells by boosting immune detection [86]. In sum, this study added to the previously identified benefits of EUG [17] and AST [87]. Their antioxidant and cardioprotective abilities against DOX toxicity have been exceeded to their post-translational modification ability through histones acetylation and immune regulation, which resulted in a significant synergism to DOX- cytotoxic effect on HR+ breast cancer cells (MCF7).

## Conclusions

In conclusion, EUG and AST enhanced the cytotoxic activity of DOX through two different apoptotic approaches, mainly through the non-MDR pathway of histones acetylation and immunonomodulation in hormone receptor positive breast cancer cells. EUG and AST significantly synergize DOX cytotoxicity in HR+ breast cancer cells. Combined use of EUG or AST with DOX significantly increased histones acetylation, Rhodamine123 uptake and caspase-3%, and decreased protein expression of aromatase, EGFR, CK7 and LC3B. DOX combined with EUG significantly increased IFNγ and decreased TNFα but vice versa was observed when AST combined with DOX where a significant reduction in IFNγ expression accompanied with TNFα overexpression was shown in comparison with DOX alone. Both EUG and AST have non-significant effect on FOXP3 mRNA expression. EUG combination caused shifting of cells from G2/M to S and G0/ G1 phases, whereas AST combination caused non-significant change. EUG combination induced apoptosis through increasing BAX/ BCl2 ratio, while AST combination was through increasing caspase- 8 expression.

Worth mentioning is that, the produced synergistic cytotoxicity of EUG and AST combined with DOX in MCF7 cells as a model of luminal A breast cancer subtype is likely could be reproduced in the other breast cancer molecular subtypes including luminal B, triple negative and Her2-enriched. Therefore, our results warrant detailed mechanistic studies to confirm the chemosensitizing effects of EUG and AST to minimize the therapeutic dose of DOX with the consequent decrease in its organ toxicity.

## Data Availability

All data analyzed in this study is available from the corresponding author on reasonable request.

## Abbreviations

DOX: Doxurobicin
EUG: Eugenol
AST: Astaxanthin
HR +: Hormonal receptor positive
EGFR: Epidermal growth factor receptor
HAT: Acetyltransferase
HDAC: Histone deacetylase
TNF: Tumour necrosis factor
IFN-γ: Interferon-γ
FOXP3: Forkhead box P3
